# The Epidemiology of Primary Biliary Cholangitis in European Countries: A Systematic Review and Meta-Analysis

**DOI:** 10.1155/2021/9151525

**Published:** 2021-06-19

**Authors:** Jakub Gazda, Sylvia Drazilova, Martin Janicko, Peter Jarcuska

**Affiliations:** 2nd Department of Internal Medicine, PJ Safarik University in Kosice and L. Pasteur University Hospital, Trieda SNP 1, Kosice 040 11, Slovakia

## Abstract

**Background:**

Primary biliary cholangitis (PBC) is a chronic autoimmune cholestatic liver disease with wide ranges of reported incidence and prevalence.

**Aim:**

To map the incidence and prevalence of PBC in European countries from 2000 through 2020.

**Methods:**

Following PRISMA recommendations, we searched the Medline and Scopus databases for studies with information on either the incidence or prevalence of PBC. After data extraction, we used a random-effects model to estimate both the pooled annual incidence rate and pooled point-prevalence rate and performed subgroup analyses to identify components contributing to between-study heterogeneity.

**Results:**

We performed a qualitative and quantitative analysis of 18 studies. The pooled point-prevalence rate was 22.27 cases per 100,000 inhabitants (95% CI: 17.98–27.01), and the pooled annual incidence rate was 1.87 new cases per 100,000 inhabitants (95% CI: 1.46–2.34). In the subgroup analyses, we proved that a small part of the between-study heterogeneity is significantly associated with a history of being part of the Eastern Bloc.

## 1. Introduction

Primary biliary cholangitis (PBC) is a chronic inflammatory autoimmune cholestatic liver disease [1]. The aetiology of PBC remains unknown; however, PBC is associated with a myriad of both HLA and non-HLA genes as well as with several environmental factors (socioeconomic status, infectious agents, environmental pollutants, vitamin D, nutrition, drugs, and physical and psychological stresses) [2]. An increased prevalence of PBC has been associated with proximity to waste disposal sites [3, 4], and in the past, it has also been associated with a north-south latitudinal gradient [5, 6]. In the USA, the prevalence increased from 2004 through 2014 despite a steady incidence [7], and the global prevalence and incidence of PBC still vary widely with geographic region. In this meta-analysis, we tried to pool the PBC incidence and prevalence reported from European countries. Furthermore, we investigated the extent to which different components may have contributed to between-study heterogeneity. A similar worldwide study and one particularly from the Asia-Pacific region have recently been reported [8, 9].

## 2. Materials and Methods

This meta-analysis was conducted and reported according to the Preferred Reporting Items for Systematic Reviews and Meta-Analyses Statement (https://www.prisma-statement.org/) [10].

### 2.1. Search Strategy

The Medline and Scopus databases were searched for studies with information on either the incidence or prevalence of PBC. The last search was run on 7 July 2020. A literature review was created using the following search terms: (“epidemiology” or “prevalence” or “incidence”) AND (“primary biliary cirrhosis” or “primary biliary cholangitis” or “autoimmune liver disease” or “sclerosing cholangitis” or “biliary liver cirrhosis”). Medical Subject Headings (MESH) were used to increase the precision and efficiency of the search. No language, publication date, or publication status restrictions were imposed. In addition, we expanded the search using the reference lists of relevant review articles identified during the search. Two authors independently screened the literature review using titles and abstracts and assessed full texts where eligible. Disagreements over the inclusion of articles were resolved by discussion with a senior hepatologist.

### 2.2. Inclusion and Exclusion Criteria

Studies were included if they met the following criteria: (1) the study was original research; (2) the study reported a prevalence or incidence (or it reported raw data that allowed the calculation of estimates); (3) the study was conducted in Europe; and (4) the study was published in 2000 or later.

Exclusion criteria for the meta-analysis were as follows: (1) the study was a review article; (2) the study was a genome study or an animal study; (3) the study described the epidemiology of PBC among hospitalized patients; and (4) the study did not specifically describe patients with PBC.

### 2.3. Data Extraction

Two investigators independently performed the data extraction. We developed a data extraction sheet, pilot-tested it on five included studies, and refined it accordingly. Furthermore, we attempted to acquire any missing information by contacting the corresponding authors of two studies; however, neither one responded to our request. Disagreements over extracted information were resolved by discussion with a senior hepatologist. The following information was extracted from each study: (1) the first author, (2) publication year, (3) country of origin, (4) case-finding methods, (5) methods of diagnosis, (6) raw data (underlying population and number of cases), and estimates of incidence and prevalence together with (7) sex-specific estimates, where available. Age-standardized estimates were preferred to crude estimates. Worth noting is that when multiple annual incidence rates were reported in a specific study, the median value for the period was calculated.

### 2.4. Statistical Analyses

The incidence and prevalence rates were adapted from the original reports. As needed, the underlying population was used to impute the number of cases and vice versa. For sex-specific analyses, the underlying population was divided by two. We used a random-effects model to estimate both the pooled annual incidence rate and the pooled point-prevalence rate (reported per 100,000 inhabitants). The results of meta-analyses are presented graphically using forest plots. We employed the DerSimonian–Laird (DL) approach to estimate the between-study heterogeneity. Two different measures of between-study heterogeneity are reported in this study: (1) *Q* is a *χ*^2^ statistic; its *p* value ≤0.05 indicates the presence of significant between-study heterogeneity, which requires further investigation, and (2) *I*^2^-statistics (inconsistency), which represents the ratio of between-study variance to the total observed variance. Outlying studies were identified by screening for externally studentized residuals that were larger than three in the absolute value. Furthermore, we assessed the possibility of publication bias by constructing funnel plots, which were assessed both visually and formally with Egger's test. We hypothesized that between-study heterogeneity could be partially associated with the inclusion of studies with different levels of risk of within-study bias. Therefore, we performed prespecified subgroup analyses and multiple metaregressions on the four following components, evaluating their effect on between-study heterogeneity: (1) the number of case-finding methods (cut-off value ≥ 2), (2) diagnostic methods (those complying with the current EASL recommendations were labelled “standard”), and (3) the underlying population (the median of the underlying populations served as the cut-off value). (4) We further investigated whether presence in the former Eastern Bloc may have contributed to different rates when compared to those reported from the former Western Bloc. Choropleth maps with colour progression were used to illustrate annual incidence rates and point-prevalence rates. In the case of multiple reports from the same country, the report based on the largest underlying population was used. All tests were two-sided and performed at the 0.05 significance level. Statistical analyses were performed in RStudio (version 1.2.1335).

## 3. Results

**Figure 1:**
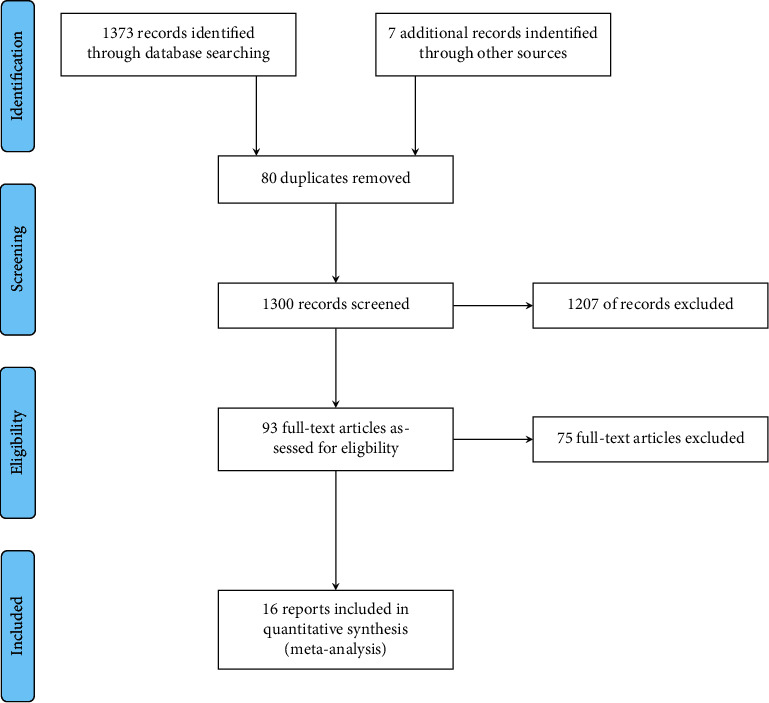
Flowchart of studies inclusion.

The electronic search yielded 1,373 records (Medline 1,200; Scopus 173). We identified seven more records reviewing the references of PBC-relevant review articles. No unpublished studies were included. After removing duplicates (*n* = 80), we screened the titles and abstracts of 1,300 records. A total of 93 reports were identified as potentially meeting our inclusion criteria and full-text articles were retrieved and examined in detail. After full-text review, 16 reports were used in subsequent meta-analysis. The PRISMA flow diagram is presented in Figure 1.

### 3.1. Studies Characteristics

A total of 16 reports on 18 different studies that were conducted in 13 European countries were included in the analysis. The publication dates of all included studies ranged from April 2007 to June 2020. A total of 17 studies (94.44%) reported local prevalence rates (10–58.2 PBC cases per 100,000 inhabitants) and 13 studies (72.22%) reported local incidence rates (0.79–5.31 new PBC cases per 100,000 inhabitants per year). Seven of these studies (38.89%) reported sex-specific rates. Furthermore, seven studies (38.89%) used at least two case-finding methods and 11 studies (61.11%) reported on specific diagnostic criteria (Table 1). A total of 25,343 cases of PBC were identified in the underlying population of 107,578,769 inhabitants.

### 3.2. Prevalence of PBC in European Countries

**Figure 2:**
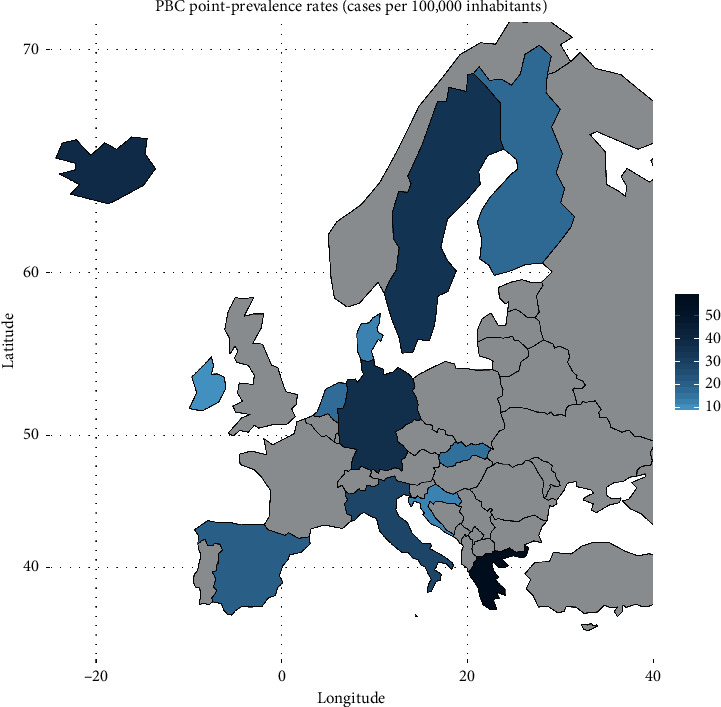
Choropleth map of PBC point-prevalence rates in Europe.

**Figure 3:**
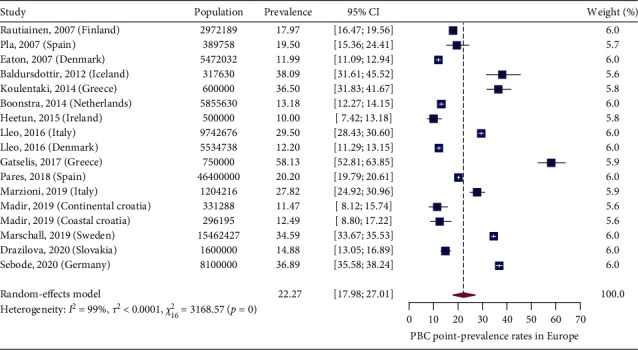
PBC point-prevalence rates in Europe.

**Figure 4:**
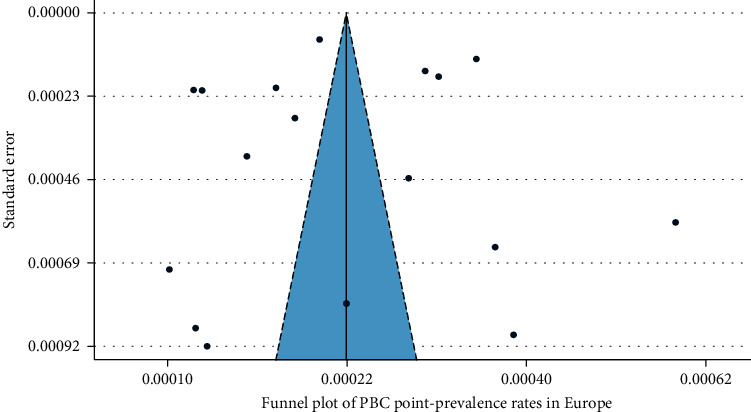
Funnel plot of PBC point-prevalence rates in Europe.

**Figure 5:**
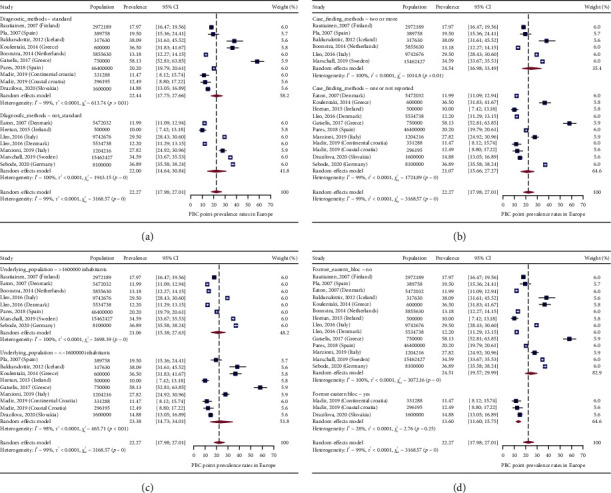
Subgroup analyses of PBC point-prevalence rates. (a) Diagnostic criteria. (b) Case-finding methods. (c) Underlying population. (d) Former Eastern/Western Bloc.

**Figure 6:**
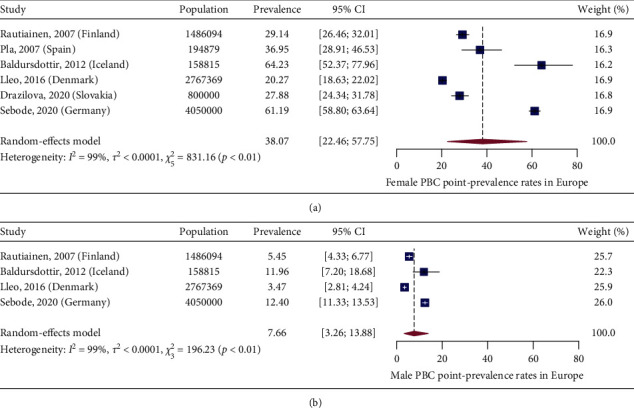
(a) Female PBC point-prevalence rates in Europe. (b) Male PBC point-prevalence rates in Europe.

In Figure 2, we present a choropleth map of European countries with a colour progression representing PBC point-prevalence rates. Meta-analytic pooling of the prevalence estimates yielded a summary point-prevalence rate of 22.27 cases per 100,000 inhabitants (95% CI: 17.98–27.01; *Q*: 3168.57, *p* < 0.0001; *I*^2^: 99%, Figure 3). The funnel plot (Figure 4) and Egger's test revealed no publication bias (*p*=0.97), and no influential studies were identified during the influential analysis. Because of significant heterogeneity, potential moderators were explored by subgroup meta-analyses (Figure 5(a)–5(d)) and a multiple metaregression. Neither the diagnostic criteria (*p* > 0.05) and the case-finding methods (*p* > 0.05) nor the underlying population (*p* > 0.05) explained the presence of heterogeneity. However, countries from the former Eastern Bloc had significantly lower point-prevalence rates when compared to those reported from the former Western Bloc (estimate: −0.0071, 95% CI: −0.0127–0.0016, *p* < 0.05). In the female population, the summary point-prevalence rate was 38.07 cases per 100,000 women (95% CI: 22.46–57.75; *Q*: 831.16, *p* < 0.01; *I*^2^: 99%; Figure 6(a)). In the male population, the summary point-prevalence rate was 7.66 cases per 100,000 men (95% CI: 3.26–13.88; *Q*:196.23, *p* < 0.01; *I*^2^: 99%; Figure 6(b)).

### 3.3. Incidence of PBC in European Countries

**Figure 7:**
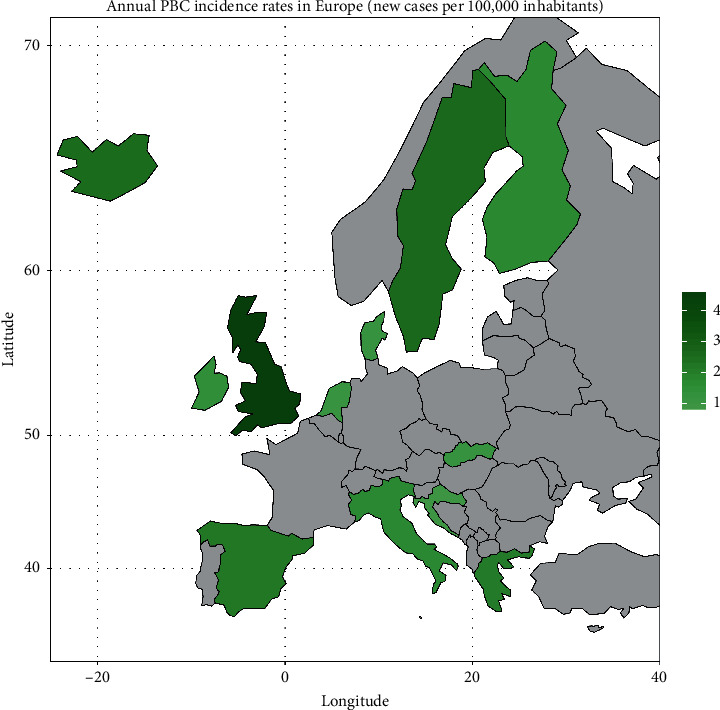
Choropleth map of annual PBC incidence rates in Europe.

**Figure 8:**
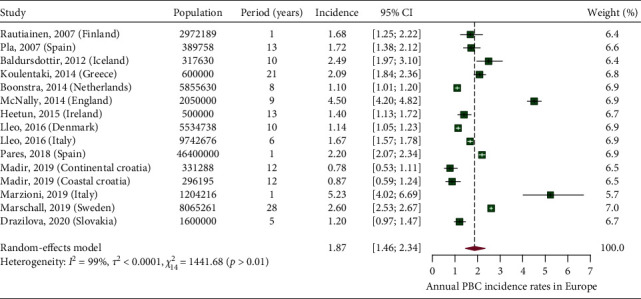
Annual PBC incidence rates in Europe.

**Figure 9:**
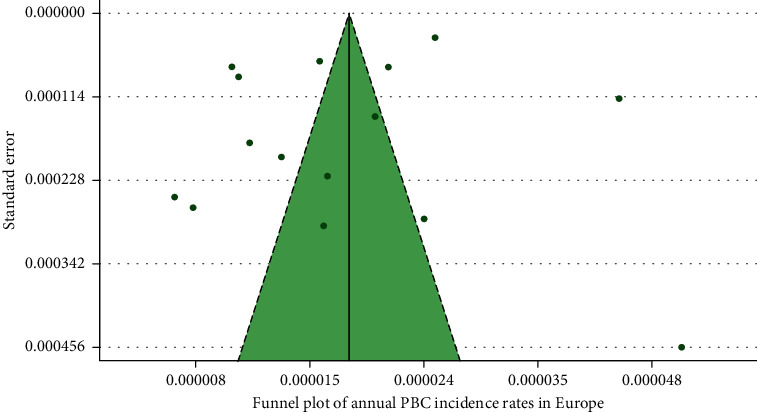
Funnel plot of annual PBC incidence rates in Europe.

**Figure 10:**
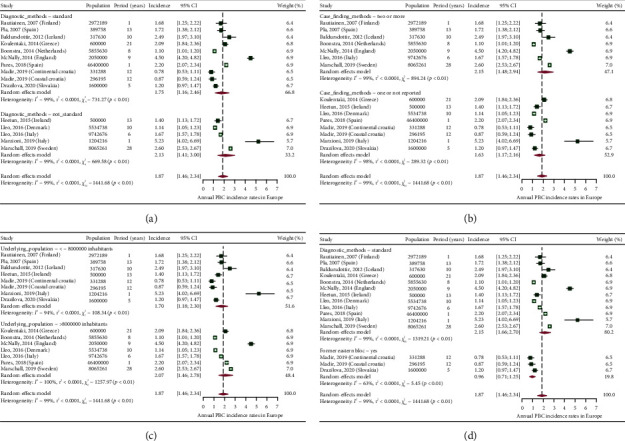
Subgroup analyses of annual PBC incidence rates. (a) Diagnostic criteria. (b) Case-finding methods. (c) Underlying population. (d) Former Eastern/Western Bloc.

**Figure 11:**
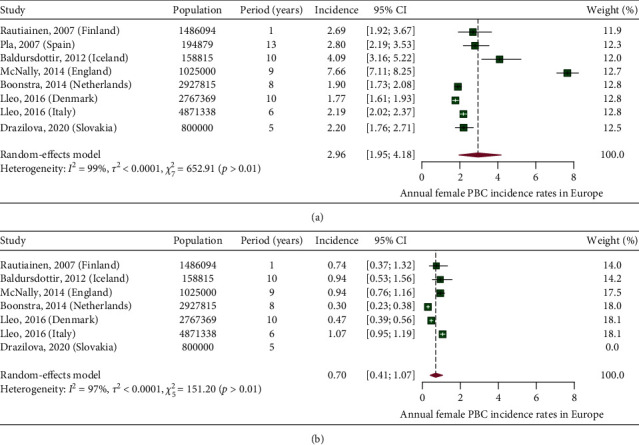
(a) Annual female PBC incidence rates in Europe. (b) Annual male PBC incidence rates in Europe.

In Figure 7, we present a choropleth map of European countries with a colour progression representing annual PBC incidence rates. Meta-analytic pooling of the annual incidence estimates yielded a summary annual incidence rate of 1.87 cases per 100,000 inhabitants (95% CI: 1.46–2.34; *Q*: 1441.68, *p* < 0.01; *I*^2^: 99%; Figure 8). The funnel plot (Figure 9) and Egger's test revealed no publication bias (*p*=0.36), and no influential studies were identified during the influential analysis. Due to strong evidence of heterogeneity, potential moderators were explored by subgroup meta-analyses (Figure 10(a)–10(d)) and simple metaregressions. However, neither the diagnostic criteria (*p* > 0.05), the case-finding methods (*p* > 0.05), the underlying population (*p* > 0.05), nor the historical presence in either of the Europe's political blocs (*p* > 0.05) explained the presence of heterogeneity. In the female population, the summary annual incidence rate was 2.96 cases per 100,000 women (95% CI: 1.95–4.18; *Q*: 652.91, *p* < 0.01; *I*^2^: 99%; Figure 11(a)). In the male population, the summary annual incidence rate was 0.70 cases per 100,000 men (95% CI: 0.41–1.07; *Q*:151.20, *p* < 0.01; *I*^2^: 99%; Figure 11(b)).

## 4. Discussion

This study aimed to map the incidence and prevalence rate of PBC in Europe. The pooled point-prevalence rate was 22.27 cases per 100,000 inhabitants (95% CI: 17.98–27.01), and the pooled annual incidence rate was 1.87 new cases per 100,000 inhabitants (95% CI: 1.46–2.34). PBC, similarly to other autoimmune disorders, is a female-predominant disease [1]. In Europe, the female prevalence was approximately five times higher compared to estimates from the male population, and the female incidence was four times higher. PBC is associated with lifestyle and both genetic and environmental factors. The population of the first-degree relatives of patients with PBC has higher prevalence of the disease when compared to the general population [27]. Smoking, several xenobiotics, oestrogen, hormonal contraception, and proximity to a toxic-waste disposal site are all associated with an increased incidence of PBC [3, 28]. An association with infectious diseases was also reported [28]. However, we did not analyse the association of these factors and the incidence or prevalence of PBC.

The employment of different case-finding methods may result in different reported rates. We found that both the prevalence (24.54, 95% CI: 16.98–33.49) and the incidence rate (2.15, 95% CI: 1.48–2.94) were higher in studies that reported at least two case-finding methods when compared to studies that did not report any case-finding method or reported only one (prevalence rate: 21.07, 95% CI: 15.66–27.27; incidence rate: 1.63, 95% CI: 1.17–2.16). However, this subgroup analysis did not explain the presence of heterogeneity.

The incidence was relatively stable during the last couple of years. The prevalence, on the other hand, steadily increased [7, 24, 25]. We will try to provide a simple explanation for this phenomenon. (1) Nowadays, awareness about PBC is getting better and diagnostic examinations are more accessible than they were in the past. (2) Advances in pharmacotherapy have resulted in lower liver-related mortality.

Few studies reported a north-south, north-west, or south-east prevalence gradient [23, 29]. Analysing choropleth maps, we did not confirm the existence of such a gradient on the European scale. We did, however, identify a lower incidence and prevalence rate of PBC in former communist states [23, 25] when compared to other European countries. We can explain this phenomenon by the worse awareness of PBC among local physicians. Likewise, Drazilova et al. described significant differences in PBC prevalence among neighbouring counties in Eastern Slovakia [25]. However, even in postcommunist countries, the prevalence is still rising [25].

The European Union, the United Kingdom, Switzerland, and Norway altogether have approximately 527 million inhabitants. When extrapolating from the pooled prevalence rate, roughly 115,000 patients should be diagnosed with PBC in these countries. However, the true number of cases would be significantly higher because a substantial portion of PBC patients, specifically patients with the asymptomatic clinical course, remains undiagnosed. According to one report, approximately one in 1,000 women could be suffering from PBC [30]. Interestingly, we described an even higher prevalence in two counties of eastern Slovakia (10% of counties), even though the overall PBC prevalence in eastern Slovakia was severalfold lower [25]. Ursodeoxycholic acid is the first-line treatment and is well accessible in the European Union [1]. Approximately 70% of patients respond partially or even completely according to the Toronto criteria [25]. The first-line treatment reduces liver-related mortality by about 50% [7]. The only second-line treatment approved by the European Medicines Agency (EMA) for the treatment of PBC is obeticholic acid (OCA), although reports on the effect of bezafibrate are promising as well [31, 32]. OCA is an expensive treatment, and good knowledge of the epidemiological situation can help estimate the cost of such a treatment on a country-wide scale. The systematic mapping of both the incidence and prevalence of PBC in the European population is the main advantage of this study. The main limitation of this study is significant between-study heterogeneity. However, we cannot confirm that this heterogeneity is due to either different case-finding methods, diagnostic criteria, or underlying populations.

## 5. Conclusion

We describe the incidence and prevalence of PBC in European countries. The true prevalence is probably higher than the reported prevalence, because asymptomatic patients are frequently undiagnosed. Improving awareness of PBC among physicians will catalyse a more effective diagnostic process and will thus result in a higher prevalence of PBC in the European population.

## Figures and Tables

**Table 1:** Reports on PBC incidence and/or prevalence from European countries.

First author	Country	Publication year	Case-finding methods	Diagnostic methods	Population	Prevalence	Female prevalence	Male prevalence	Incidence	Female incidence	Male incidence
Rautiainen et al. [11]	Finland	2007	1, 2, 5, 6	2/3 of a, b, d	2,972,189	18.0	29.2	5.5	1.7	2.7	0.8
Pla et al. [12]	Spain	2007	1, 2, 3, 5	2/3 of a, b/c, d	389,758	19.5	37.02	—	1.72	2.84	—
Eaton et al. [13]	Denmark	2007	2	ICD	5,472,032	12.0	—	—	—	—	—
Baldursdottir et al. [14]	Iceland	2012	1, 2, 3, 5	2/3 of a, b, d	317,630	38.3	64.4	12.5	2.5	4.1	1.0
McNally et al. [15]	England	2014	1, 2, 3, 4, 5	2/3 of a, b, d	2,050,000	—	—	—	4.509	7.668	0.949
Koulentaki et al. [16]	Greece	2014	2	2/3 of a, b, d	600,000	36.5	—	—	2.088	—	—
Boonstra et al. [17]	Netherlands	2014	1, 3, 5, 6, 7	2/3 of a, b, d	5,855,630	13.2	—	—	1.1	1.9	0.3
Heetun et al. [18]	Ireland	2015	2	—	500,000	10.0	—	—	1.4	—	—
Lleo et al. [19]	Italy	2016	2, 7	—	9,742,676	29.5	—	—	1.67	2.19	1.07
Lleo et al. [19]	Denmark	2016	2	—	5,534,738	12.2	20.3	3.5	1.14	1.77	0.47
Gatselis et al. [20]	Greece	2017	8	2/3 of a, b/c, d	750,000	58.2	—	—	—	—	—
Pares et al. [21]	Spain	2018	1 (delphi)	2/3 of a, b, d/US	46,400,000	20.2	—	—	2.2	—	—
Marzioni et al. [22]	Italy	2019	2	ICD	1,204,216	27.9	—	—	5.31	—	—
Madir et al. [23]	Croatia	2019	2	2/3 of a, b, d	331,288	11.5	—	—	0.79	—	—
Madir et al. [23]	Croatia	2019	2	2/3 of a, b, d	296,195	12.5	—	—	0.89	—	—
Marschall et al. [24]	Sweden	2019	2, 4, 7	ICD	8,065,261	34.6	—	—	2.6	—	—
Drazilova et al. [25]	Slovakia	2020	1	2/3 of a, b/c, d	1,600,000	14.9	28.0	—	1.2	2.2	—
Sebode et al. [26]	Germany	2020	7	ICD	8,100,000	36.9	61.2	12.4	—	—	—

The point-prevalence rate is reported as cases per 100,000 inhabitants. The annual incidence rate is reported as new cases per 100,000 inhabitants. Cases-finding methods: (1) survey of physicians, (2) hospital records, (3) laboratory data on antimitochondrial antibody positivity, (4) death notifications, (5) histology data on liver biopsies, (6) liver transplant records, (7) pharmacy or insurance databases or billing system, and (8) prospectively collected registry. Diagnostic methods: (a) cholestatic liver panel, (b) antimitochondrial antibody positivity, (c) antinuclear (anti-gp210/anti-sp100) antibody positivity, (d) compatible liver histology, ICD: International Classification of Diseases, US: abdominal ultrasound.

## Data Availability

The data (in an excel file) used to support the findings of this study are available from the corresponding author upon request.

## References

[1] European Association for the Study of the Liver (2017). EASL clinical practice guidelines: the diagnosis and management of patients with primary biliary cholangitis. *Journal of Hepatol*.

[2] Lleo A., Leung P. S., Hirschfield G. M., Gershwin E. M. (2020). The pathogenesis of primary biliary cholangitis: a comprehensive review. *Semin Liver Disease*.

[3] Ala A., Stanca C. M., Bu-Ghanim M. (2006). Increased prevalence of primary biliary cirrhosis near superfund toxic waste sites. *Hepatology*.

[4] Smyk D., Mytilinaiou M. G., Rigopoulou E. I., Bogdanos D. P. (2010). PBC triggers in water reservoirs, coal mining areas and waste disposal sites: from newcastle to New York. *Disease Markers*.

[5] Terziroli Beretta-Piccoli B., Stirnimann G., Cerny A. (2018). Geoepidemiology of primary biliary cholangitis: lessons from Switzerland. *Clinical Reviews in Allergy & Immunology*.

[6] Selmi C., Gershwin M. E. (2009). The role of environmental factors in primary biliary cirrhosis. *Trends in Immunology*.

[7] Lu M., Zhou Y., Haller I. V. (2018). Increasing prevalence of primary biliary cholangitis and reduced mortality with treatment. *Clinical Gastroenterology and Hepatology*.

[8] Lv T., Chen S., Li M., Zhang D., Kong Y., Jia J. (2020). Regional variation and temporal trend of PBC epidemiology: a systematic review and meta‐analysis. *Journal of Gastroenterology and Hepatology*.

[9] Zeng N., Duan W., Chen S. (2019). Epidemiology and clinical course of primary biliary cholangitis in the Asia-Pacific region: a systematic review and meta-analysis. *Hepatology International*.

[10] Liberati A., Altman D. G., Tetzlaff J. (2009). The PRISMA statement for reporting systematic reviews and meta-analyses of studies that evaluate health care interventions: explanation and elaboration. *Journal of Clinical Epidemiology*.

[11] Rautiainen H., Salomaa V., Niemelä S. (2007). Prevalence and incidence of primary biliary cirrhosis are increasing in Finland. *Scandinavian Journal of Gastroenterology*.

[12] Pla X., Vergara M., Gil M. (2007). Incidence, prevalence and clinical course of primary biliary cirrhosis in a Spanish community. *European Journal of Gastroenterology & Hepatology*.

[13] Eaton W. W., Rose N. R., Kalaydjian A., Pedersen M. G., Mortensen P. B. (2007). Epidemiology of autoimmune diseases in Denmark. *Journal of Autoimmunity*.

[14] Baldursdottir T. R., Bergmann O. M., Jonasson J. G., Ludviksson B. R., Axelsson T. A., Björnsson E. S. (2012). The epidemiology and natural history of primary biliary cirrhosis. *European Journal of Gastroenterology & Hepatology*.

[15] McNally R. J. Q., James P. W., Ducker S., Norman P. D., James O. F. W. (2014). No rise in incidence but geographical heterogeneity in the occurrence of primary biliary cirrhosis in North East England. *American Journal of Epidemiology*.

[16] Koulentaki M., Mantaka A., Sifaki-Pistolla D., Thalassinos E., Tzanakis N., Kouroumalis E. (2014). Geoepidemiology and space-time analysis of primary biliary cirrhosis in Crete, Greece. *Liver International*.

[17] Boonstra K., Kunst A. E., Stadhouders P. H. (2014). Rising incidence and prevalence of primary biliary cirrhosis: a large population-based study. *Liver International*.

[18] Heetun Z., Maher N., Buggy A., Carroll P., Aftab A., Courtney G. (2015). Prevalence and epidemiology of autoimmune hepatitis and primary biliary cirrhosis across the South-Eastern regional health board. *Proceedings of the Irish Journal of Medical Science*.

[19] Lleo A., Jepsen P., Morenghi E. (2016). Evolving trends in female to male incidence and male mortality of primary biliary cholangitis. *Scientific Reports*.

[20] Gatselis N. K., Zachou K., Lygoura V. (2017). Geoepidemiology, clinical manifestations and outcome of primary biliary cholangitis in Greece. *European Journal of Internal Medicine*.

[21] Parés A., Albillos A., Andrade R. J. (2018). Primary biliary cholangitis in Spain. Results of a Delphi study of epidemiology, diagnosis, follow-up and treatment. *Revista espanola de enfermedades digestivas*.

[22] Marzioni M., Bassanelli C., Ripellino C., Urbinati D., Alvaro D., Disease L. (2019). Epidemiology of primary biliary cholangitis in Italy: evidence from a real-world database. *Digestive and Liver Disease*.

[23] Madir A., Božin T., Mikolašević I. (2019). Epidemiological and clinical features of primary biliary cholangitis in two croatian regions: a retrospective study. *Croatian Medical Journal*.

[24] Marschall H. U., Henriksson I., Lindberg S. (2019). Incidence, prevalence, and outcome of primary biliary cholangitis in a nationwide Swedish population-based cohort. *Scientific Reports*.

[25] Drazilova S., Babinska I., Babinska I. (2020). Epidemiology and clinical course of primary biliary cholangitis in Eastern Slovakia. *International Journal of Public Health*.

[26] Sebode M., Kloppenburg A., Aigner A., Lohse A. W., Schramm C., Linder R. (2020). Population-based study of autoimmune hepatitis and primary biliary cholangitis in Germany: rising prevalences based on ICD codes, yet deficits in medical treatment. *Zeitschrift für Gastroenterologie*.

[27] Corpechot C., Chrétien Y., Chazouillères O., Poupon R. (2010). Demographic, lifestyle, medical and familial factors associated with primary biliary cirrhosis. *Journal of Hepatology*.

[28] Kouroumalis E. (2010). Environmental agents involved in the cause of primary biliary cirrhosis. *Disease Markers*.

[29] Rigopoulou E. I., Davies E., Pares A. (2005). Prevalence and clinical significance of isotype specific antinuclear antibodies in primary biliary cirrhosis. *Gut*.

[30] Jepsen P., Grønbæk L., Vilstrup H. (2015). Worldwide incidence of autoimmune liver disease. *Digestive Diseases*.

[31] Nevens F., Andreone P., Mazzella G. (2016). A placebo-controlled trial of obeticholic acid in primary biliary cholangitis. *New England Journal of Medicine*.

[32] Corpechot C., Chazouillères O., Rousseau A. (2018). A placebo-controlled trial of bezafibrate in primary biliary cholangitis. *New England Journal of Medicine*.

